# Troxerutin protects hippocampal neurons against amyloid beta-induced oxidative stress and apoptosis

**DOI:** 10.17179/excli2017-526

**Published:** 2017-08-09

**Authors:** Fereshteh Farajdokht, Mohammad Amani, Fariba Mirzaei Bavil, Alireza Alihemmati, Gisou Mohaddes, Shirin Babri

**Affiliations:** 1 Neurosciences Research Center (NSRC), Tabriz University of Medical Sciences, Tabriz, Iran; 2 Drug Applied Research Center of Tabriz University of Medical Sciences, Tabriz, Iran

**Keywords:** Alzheimer's disease, amyloid beta, acetylcholinesterase, oxidative stress

## Abstract

Alzheimer's disease (AD) is an age-related neurodegenerative disease linked with increased production and/or deposition of amyloid-beta (Aβ) in the brain. The aim of the present study was to investigate the possible neuroprotective effect of troxerutin on an animal model of Alzheimer's disease. Alzheimer model was induced by a single dose intracerebroventricular (ICV) injection of Aβ 1-42 (5 nmol/5 µl). Thereafter, troxerutin (300 mg/kg) was gavaged for 14 days. The hippocampal malondialdehyde (MDA) levels and enzymatic activities of superoxide dismutase (SOD), glutathione peroxidase (GPx), and acetylcholinesterase (AChE) were measured using enzyme-linked immunosorbent assay (ELISA) method. In addition, the number of apoptotic cells in the dentate gyrus (DG) was assessed by TUNEL kit. The results showed that ICV microinjection of Aβ 1-42 increased MDA levels, reduced SOD and GPx, and increased AChE activities in the hippocampus. Chronic administration of troxerutin significantly attenuated MDA levels and AChE activity and increased SOD and GPx activities in the hippocampus. Moreover, the number of apoptotic cells was decreased by troxerutin treatment. Taken together, our study demonstrated that troxerutin could increase the resistance of hippocampal neurons against apoptosis, at least in part, by diminishing the activity of AChE and oxidative stress. Therefore, troxerutin may have beneficial effects in the management of Alzheimer's disease.

## Introduction

Alzheimer's disease (AD) is a neurodegenerative disease characterized by progressive loss of memory and cognitive function (Butterfield and Boyd-Kimball, 2004[9]). AD is the most common cause of dementia in the people over 65 years which imposes a significant economic burden on families and society, and remarkably decreases the quality of life (Takizawa et al., 2015[54]).

Two main neurological hallmarks in AD are extracellular senile plaques and intracellular neurofibrillary tangles in the brain regions critical for learning and memory, the hippocampus and other cortices, resulting in the loss of neurons and synapses (Blennow and Hampel, 2003[7]; Giannakopoulos et al., 2003[20]; Hardy and Selkoe, 2002[22]). Although, the exact mechanism of AD remains unclear, it seems that alterations in the production and processing of Aβ leads to accumulation of Aβ plaques in the neuronal space of the brain (Bloom, 2014[8]; Mayeux and Stern, 2012[33]). Other proposed mechanisms associated with AD are mitochondrial dysfunction and oxidative stress (Pohanka, 2014[46]; Wang et al., 2014[56]), impairment of cholinergic transmission (Kumar and Singh, 2015[29]), neuro-inflammation (Morales et al., 2014[34]), and glutamate neurotoxicity (Rudy et al., 2015[50]).

Oxidative stress is defined as an imbalance between pro-oxidant stress and anti-oxidant defense which may lead to tissue injury (Halliwell and Gutteridge, 1999[21]). Previous studies support the vulnerability of the central nervous system (CNS) to oxidative stress possibly due to large rate of oxygen consumption, the richness of iron, high level of polyunsaturated fatty acids, and low levels of antioxidants (Butterfield et al., 2001[10]; Paula et al., 2005[42]). Recent studies have highlighted the importance of oxidative processes in the pathogenesis of AD (Cioanca et al., 2015[13]; E Abdel Moneim, 2015[15]). Although the initiating events are still unknown, it has been proposed that oxidative damage is involved in the initiation of AD and is the first apparent sign in progression of AD (Arimon et al., 2015[1]; Wang et al., 2014[56]). Antioxidant enzymes including superoxide dismutase (SOD), thioredoxin, glutathione peroxidase (GPx), glutathione reductase (GR), and catalase (CAT) form important protective mechanism against reactive oxygen species (ROS) (Birben et al., 2012[6]; Pohanka, 2014[46]). Previous studies have shown that the activities of antioxidant enzymes are diminished, whereas the levels of oxidative stress markers are elevated in the brain of AD patients (Arimon et al., 2015[1]; Krstic and Knuesel, 2013[28]).

Acetylcholine (ACh) and cholinergic system are essential for regulation of learning and memory processes (Papandreou et al., 2011[40]). Previous studies showed that accumulation of Aβ reduced ACh levels in the AD brain through increasing the expression of AChE (Perry et al., 1992[44]). Moreover, AChE has the capability to augment Aβ deposition and fibril formation (Chacón et al., 2003[11]). Under normal conditions AChE is not an apoptosis initiator; however overexpression of AChE increases the sensitivity of cells to apoptosis (Zhang and Greenberg, 2012[59]). Although several agents such as cholinesterase inhibitors (Parsons et al., 2013[41]), M1 muscarinic receptor agonists (Jiang et al., 2014[25]), and some of phosphodiesterases (Fiorito et al., 2013[18]) are used to relieve symptoms of AD, most of these drugs are toxic and have numerous side effects. 

Moreover, there is an inverse relationship between oxidative stress and Aβ levels. Persson et al. indicated that oxidative stress increased production and accumulation of Aβ, which in turn increased ROS production and mitochondrial dysfunction (Persson et al., 2014[45]). Several studies found that diminished number of neurons and synapses due to neuronal apoptosis in the cerebral cortex and hippocampus is the main reason of cognitive impairment of AD (Morishima et al., 2001[36]; Scheff et al., 2006[51]). Since oxidative stress is a part of normal aging and starts very early in the disease progression, preventive therapies using antioxidants still hold great promise (Chakrabarti et al., 2014[12]). 

Troxerutin, derivative of natural bioflavonoid rutin, is found in tea, coffee, cereals, and a variety of fruits and vegetables. Troxerutin possess biological properties such as antioxidant (Panat et al., 2016[39]) and anti-inflammatory effects (Fan et al., 2009[17]). Previously we demonstrated that oral administration of troxerutin improved synaptic failure (Babri et al., 2014[3]) and learning and memory impairments induced by ICV injection of Aβ (Babri et al., 2012[2]). 

The aim of the present study was to investigate the effect of troxerutin on the hippocampal activity of AChE and oxidative status, and the number of neuronal apoptotic cells in the DG in Aβ 1-42-induced AD model in rats.

## Materials and Methods

### Animals

Sixty four adult male Wistar rats about 14 weeks old, weighing 300 to 350 g were obtained from Pasteur Institute of Iran and kept at standard conditions four per cage, 22-24 °C, 12 h light-dark cycle, and free access to food and water. All experiments were performed in agreement with guidelines of the Tabriz University of Medical Sciences for care and use of laboratory animals. After one week of habituation animals were randomly allocated into the following groups (n=12 per each group):

1. Sham operated
2. Reverse Aβ 42-1 (Bachem, Switzerland)
3. Aβ 1-42 (Bachem, Switzerland)
4. Aβ 1-42 + troxerutin (Merck, Germany).

### Surgical procedures

In order to perform stereotaxic surgery, animals were deeply anesthetized by an intraperitoneal (i.p) injection of ketamine (80 mg/ kg) and xylazine (12 mg/kg) and placed on a stereotaxic instrument (Stoelting Co., Illinois, USA). The scalp was incised and a small hole was drilled at a proper location according to the Paxinos and Watson rat brain atlas (Paxinos, 2007[43]). Aβ (5 nmol/5 µl), reverse Aβ (5 nmol/5 µl), or saline (5 µl) were injected into the right lateral ventricle (AP: −0.8, ML: 1.6 and DV: 3.5 mm below dura) using a Hamilton micro syringe during 5 min. Needle was left in the place for 5 min before it was slowly withdrawn. Animals in the Aβ + troxerutin group received troxerutin (300 mg/ kg P.O for 14 days) one hour before injection of Aβ (Babri et al., 2012[2]) and continued daily for 14 days.

### Assessment of hippocampal MDA levels and enzymatic activities of SOD, GPx and AChE

At the end of experiments, rats were deeply anesthetized with 80 mg/kg sodium pentobarbital and sacrificed by decapitation, then hippocampal tissues were immediately removed. All samples were kept at -80 °C for later analysis. Samples were homogenized in 1.15 % KCl solution and centrifuged at 1000 rpm for 1 min at 4 °C for acquiring the supernatant. The supernatants were used for determination of MDA levels, and activities of SOD, GPx, and AChE. Hippocampal MDA level was measured using the thiobarbituric acid reactive substances (TBARS) method at 535 nm with a UV spectrophotometer (Kaya et al., 2004[27]). SOD, GPx, and AChE activities were measured using the commercial rat-specific ELISA kits (Randox Crumlin, UK) according to the manufacturer's protocols and expressed as U/mg protein and nmol/mg protein in tissue homogenate.

### Histological study

Following deep anesthesia (80 mg/kg sodium pentobarbital) animals were perfused transcardially through the ascending aorta with 10-20 ml saline followed by 200 ml of 4 % paraformaldehyde. The brain tissue was removed and post fixed in the same solution, then processed for histological assay. Paraffin embedded brain tissue was cut in 10 μm coronal sections using a microtome. Brain sections were stained with TUNEL staining kit for determination of apoptotic cells in the dentate gyrus (DG) according to the manufacturer's instructions. The numbers of apoptotic cells were counted by a blind person to the treatments using a light microscope (Nikon, Tokyo, Japan) at final magnification 400×. At least average TUNEL-positive cells of eight sequential brain sections from each animal were used for analysis. 

### Statistical analysis

Data were expressed as mean ± standard error of means (S.E.M.). Data were analyzed using SPSS (version 16) with One-way ANOVA followed by Tukey post-hoc test. Data of the histological changes were analyzed by the Kruskal-Wallis test followed by the post hoc Mann-Whitney test. Significance was assessed at the p<0.05 level.

## Results

### Troxerutin attenuated hippocampal MDA levels 

**Figure 1:**
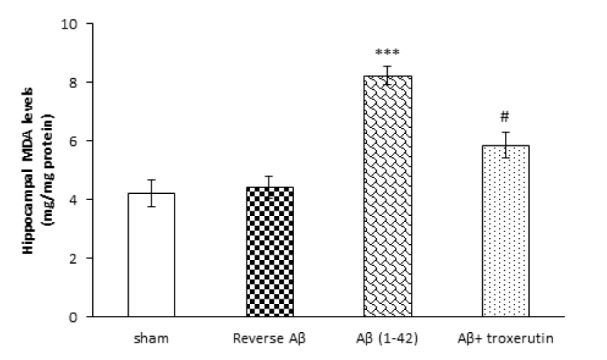
Effect of troxerutin on the hippocampal malondialdehyde (MDA) levels. Data are expressed as mean ± SEM for n=8 animals per group. *** p<0.001 vs. sham group and ^#^ p<0.05 vs. Aβ group.

To investigate the effect of chronic troxerutin treatment on oxidative stress, hippocampal MDA level was measured (Figure 1(Fig. 1)). Our results demonstrated that the level of MDA, an indicator of lipid peroxidation, in the hippocampus was significantly (p<0.001) increased by Aβ 1-42 administration, while administration of reverse Aβ 42-1 had no significant effect on MDA levels. On the other hand, treatment with troxerutin significantly (p<0.05) decreased MDA levels compared with Aβ treated animals. 

### Troxerutin enhanced antioxidant enzyme activities in the hippocampus

**Figure 2:**
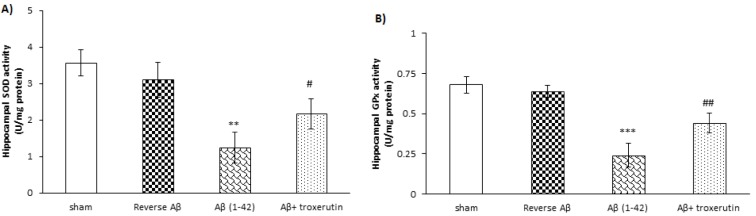
Effect of troxerutin on (A) superoxide dismutase (SOD) and (B) glutathione reductase (GPx) activities in the hippocampus. Data are expressed as mean ± SEM for n=8 animals per group.**p<0.01, *** p<0.001 vs. the sham group and ^#^ p<0.05, ^##^ p<0.01 vs. the Aβ group.

The results revealed that Aβ 1-42 administration significantly decreased the activities of SOD (p<0.01) and GPx (p<0.001), indicators of antioxidant defense, in the hippocampus (Figure 2A(Fig. 2) and 2B(Fig. 2), respectively). However, reverse Aβ 42-1 had no significant effects on the hippocampal SOD and GPx activities. Conversely, SOD (p<0.05) and GPx (p<0.01) activities were significantly increased in the chronic troxerutin treated group as compared to the Aβ-received group. 

### Troxerutin reduced the activity of AChE in the hippocampus

**Figure 3:**
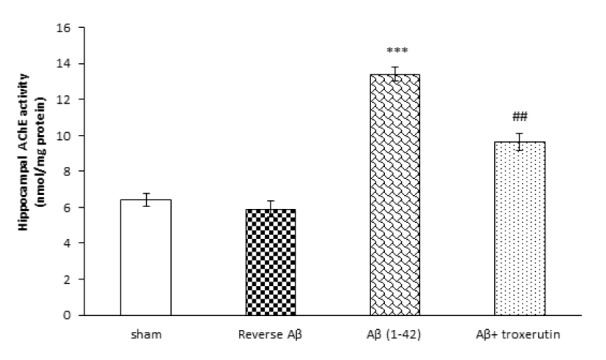
Effect of troxerutin treatment on the hippocampal acetylcholinesterase (AChE) activity. Values are expressed as the mean ± SEM for n=8 animals per group: ***p<0.001 vs. sham, ^##^ p<0.01 vs. Aβ group.

In the current study, hippocampal activity of AChE, as a cholinergic marker, was also assessed. The one-way ANOVA analysis revealed that Aβ 1-42 administration induced a significant (p<0.001) increase in the AChE activity (Figure 3(Fig. 3)). Nevertheless, treatment with troxerutin remarkably (p<0.01) attenuated the hippocampal AChE levels. Reverse Aβ treatment did not significantly affect the hippocampal AChE levels.

### Troxerutin reduced the numbers of TUNEL-positive cells in the dentate gyrus

**Figure 4:**
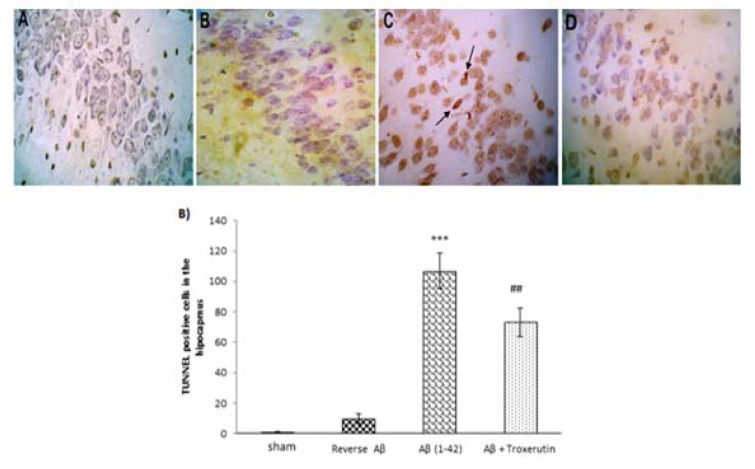
Troxerutin prevents Aβ (1-42)-induced apoptosis in the dentate gyrus (DG). (A) TUNEL staining was used to identify apoptotic nuclei in response to Aβ administration (×400) [A: sham; B: Reverse Aβ; C: Aβ (1-42); D: Aβ + troxerutin]. Central injection of Aβ induced neuronal apoptosis (black arrows) in the DG (B) TUNEL-positive cell counts. Following β-amyloid injection, an increased number of apoptotic cells were found in the dentate gyrus. Values are expressed as the mean ± SEM (n=4): ***p<0.001 vs. sham group, ^##^ p<0.01 vs. Aβ group.

Figure 4A(Fig. 4) shows the morphological features of TUNEL-stained hippocampal sections. Histological study demonstrated that DG neurons were almost intact in the sham group; however, Aβ 1-42 administration increased neuronal damage. Intriguingly, troxerutin treatment could decrease neuronal apoptosis induced by Aβ in rats.

The results of Kruskal-Wallis analysis showed that the number of TUNEL-positive cells in the DG were significantly (p<0.001) increased in the Aβ-received group (Figure 4B(Fig. 4)). In contrast, neurons were significantly (p<0.01) preserved in the troxerutin-treated group and sparse TUNEL-positive cells were found in the DG region of the hippocampus. No significant difference was observed between the sham and reverse Aβ-treated groups.

## Discussion

The present study showed that ICV injection of Aβ 1-42 increased hippocampal MDA and AChE levels and attenuated antioxidant enzymes activities (SOD and GPx). On the other hand, chronic troxerutin treatment for 14 days significantly reduced MDA levels and AChE activity, and improved enzymatic antioxidant defense in the hippocampus. Moreover, troxerutin showed a neuroprotective effect and reduced the number of TUNEL-positive cells in the DG.

Emerging evidence suggests that oxidative damage plays a causal role in the pathogenesis of AD (E Abdel Moneim, 2015[15]). Principal manifestation of oxidative stress in the CNS is lipid peroxidation occurring in the early phase of AD (Mattson, 2004[32]; Qin et al., 2009[47]). Lipid peroxidation, in part, accounts for apoptosis and neurodegeneration in the AD brain. It has also been revealed that Aβ 1-42 can lead to lipid peroxidation and neuronal apoptosis (Butterfield et al., 2001[10]; Ivins et al., 1999[24]).

In view of the fact that oxidative stress and impaired cholinergic system play a pathogenic role in AD, we investigated the effects of troxerutin on oxidative status in the hippocampus. Central injection of Aβ 1-42 provokes several impairments including oxidative stress (Bagheri et al., 2011[4]), cholinergic dysfunction (Olariu et al., 2001[38]), and neuronal apoptosis (Ivins et al., 1999[24]; Ruan et al., 2010[49]) possibly through induction of protein oxidation and lipid peroxidation. In line with other studies, our results demonstrated that Aβ 1-42 increased oxidative stress in the hippocampus which was confirmed by diminished enzymatic antioxidant defense and increased MDA levels, end product of lipid peroxidation (Butterfield and Boyd-Kimball, 2004[9]; Cioanca et al., 2013[14], 2015[13]; Turunc Bayrakdar et al., 2014[55]). Nevertheless, we found that administration of troxerutin (300 mg/kg) could significantly reverse MDA levels and enhance enzymatic antioxidant defense against Aβ 1-42 in the hippocampus.

Acetylcholine, which involves in learning and memory processes, is degraded by AChE (Papandreou et al., 2011[40]). In the AD brain, cholinergic activity decreases possibly due to increased activity of AChE around β-amyloid plaques (Moran et al., 1993[35]). It is well known that increased AChE activity within and around amyloid plaques increases cytotoxicity by promoting the aggregation of amyloid beta-peptides into fibrils which is more toxic than Aβ fibrils (Chacón et al., 2003[11]; Inestrosa et al., 2008[23]; Reyes et al., 2004[48]). Previous studies have also revealed that hyperactivity of AChE leads to memory deficit, and AChE inhibitors are effectively used for relieving symptoms of AD in rodents (Ballard et al., 2005[5]; Giacobini, 2004[19]). In the present study, Aβ noticeably increased the hippocampal AChE activity, which was in accordance with previous study (Xu et al., 2017[57]). Nevertheless, chronic troxerutin administration effectively decreased AChE activity in the hippocampus induced by Aβ. Similarly, previous study has shown that troxerutin inhibits activity of AChE in the basal forebrain, hippocampus, and frontal cortex of D-galactose-treated mice (Lu et al., 2010[30]). Furthermore, it has been shown that oxidative stress is related to the brain AChE activity (Inestrosa et al., 2008[23]). Therefore, the reduction of the hippocampal AChE activity suggests that troxerutin is capable of improving memory impairment and oxidative stress induced by Aβ 1-42. 

The present study demonstrated that Aβ 1-42 administration increased the number of TUNEL-positive cells in the DG. In support of our study, extensive evidence shows that accumulation of Aβ triggers neuronal apoptosis in the hippocampus, especially in the DG, which results in neuronal loss (Kadowaki et al., 2005[26]; Obulesu and Lakshmi, 2014[37]; Shimohama, 2000[52]; Stadelmann et al., 1999[53]; Yu et al., 2006[58]). On the other hand, several studies indicated a protective role for troxerutin in different tissues through inhibition of the oxidative stress markers (Elangovan and Pari, 2013[16]; Fan et al., 2009[17]; Lu et al., 2010[30]; Zhang et al., 2009[60]). Our results further showed that troxerutin reduced the TUNEL-positive cell counts in the DG indicating a marked inhibitory effect on cell apoptosis. Similarly, Lu et al. showed that troxerutin inhibited endoplasmic reticulum stress-induced apoptosis in the hippocampus of mice (Lu et al., 2011[31]). Therefore, troxerutin might reverse Aβ-induced neuronal loss through decreasing lipid peroxidation end product (MDA), inhibiting AChE activity, and enhancing the enzymatic antioxidant defense in the hippocampus. 

Overall the findings of the present study revealed that troxerutin attenuates Aβ 1-42-induced deleterious effects in the hippocampus of rats. This is the first study showing the neuroprotective potential of troxerutin against Aβ 1-42-induced Alzheimer's disease possibly through its anti-apoptotic, antioxidant, and AChE inhibitory effects in the hippocampus. 

## Acknowledgements

This work was financially supported by grant No. 89-60-12 from the Neurosciences Research Center (NSRC) at Tabriz University of Medical Sciences.

## Conflict of interest

None of the authors has any conflict of interest.
